# PEGylated Recombinant Adenosine Deaminase Maintains Detoxification and Lymphocyte Counts in Patients with ADA-SCID

**DOI:** 10.1007/s10875-022-01426-y

**Published:** 2023-02-25

**Authors:** Morna J. Dorsey, Arye Rubinstein, Heather Lehman, Tracy Fausnight, Joseph M. Wiley, Elie Haddad

**Affiliations:** 1 Pediatric Immunology and Allergy Center, University of California San Francisco Medical School, San Francisco, CA USA; 2 Department of Pediatrics, Montefiore Medical Center, Albert Einstein College of Medicine, Bronx, NY USA; 3 Department of Pediatrics, University of Buffalo Jacobs School of Medicine and Biomedical Sciences, Buffalo, NY USA; 4 Department of Pediatrics, Penn State Health Hershey Medical Center, Hershey, PA USA; 5 Medical Affairs, Leadiant Biosciences, Inc, Gaithersburg, MD USA; 6 Department of Pediatrics, University of Montreal, Montreal, QC Canada

**Keywords:** ADA-SCID, Enzyme replacement therapy, Elapegademase, Metabolic detoxification, Lymphocyte counts, Anti-drug antibodies

## Abstract

**Purpose:**

Metabolic detoxification with enzyme replacement therapy (ERT) promotes immune recovery in patients with adenosine deaminase (ADA)–deficient severe combined immunodeficiency (ADA-SCID). Elapegademase is a PEGylated recombinant bovine ADA ERT developed to replace the now-discontinued bovine-derived pegademase. This study was a 1-way crossover from pegademase to elapegademase in 7 patients with ADA-SCID to assess efficacy and safety outcomes for elapegademase.

**Methods:**

After once-weekly pegademase dosage was adjusted to achieve therapeutic metabolic detoxification and trough ADA activity, patients transitioned to a bioequivalent dose of elapegademase. Maintenance of metabolic detoxification and adequate ADA activity were evaluated periodically.

**Results:**

One patient withdrew after 2 doses of an early elapegademase formulation due to injection-site pain caused by EDTA. The 6 remaining patients completed 71−216 weeks of elapegademase therapy with a formulation that did not contain EDTA. In these patients, elapegademase improved ADA activity compared with pegademase and maintained metabolic detoxification. Total lymphocyte counts increased for all completer patients from between 1.2- and 2.1-fold at the end of study compared with baseline. Elapegademase had a comparable safety profile to pegademase; no patient developed a severe infectious complication. Three patients had transient, non-neutralizing antibodies to pegademase, elapegademase, and/or polyethylene glycol ≤ 47 weeks of treatment without effect on trough plasma ADA activity or trough erythrocyte deoxyadenosine nucleotide levels.

**Conclusion:**

Elapegademase was safe, well tolerated, achieved stable trough plasma ADA activity with weekly dosing, was effective in maintaining metabolic detoxification, and was associated with maintenance or improvements in lymphocyte counts compared with pegademase therapy in patients with ADA-SCID.

**Supplementary Information:**

The online version contains supplementary material available at 10.1007/s10875-022-01426-y.

## Introduction

Adenosine deaminase (ADA)–deficient severe combined immunodeficiency (ADA-SCID) is a rare, autosomal recessive, systemic, metabolic condition [1] that is usually fatal if left untreated [2, 3]. Globally, ADA-SCID is estimated to occur in approximately 1 in 200,000 to 1,000,000 newborns [2, 4] and accounts for approximately 10−15% of all SCID cases [5, 6].

Mutations in the *ADA* gene on chromosome 20q, which encodes a key enzyme of the purine salvage pathway, results in a systemic deficiency in ADA expression [2, 3, 6, 7]. To date, > 70 *ADA* mutations have been found in patients with ADA-SCID [6]. The age of onset and severity of the disease are related to expressed ADA activity, which is strongly associated with the sum of ADA activity provided by both alleles [8, 9]. Reductions in ADA expression and activity result in the accumulation of 2′-deoxyadenosine (dAdo) and its phosphorylated derivative (deoxyadenosine nucleotide [dAXP]) to toxic levels in lymphocytes and nonimmune cells [3, 6]. The disease typically manifests with profound lymphopenia (i.e., deficiencies in T, B, and NK cells) with absent or severely impaired cellular and humoral immune function; this profound lymphopenia results in severe and recurrent infections, including opportunistic infections often beginning soon after birth [6, 7]. Other clinical manifestations include failure to thrive, metabolic abnormalities (i.e., deafness, skeletal disorders, alveolar proteinosis, and neurodevelopmental issues), and a range of neurological disorders (i.e., sensorineural hearing loss, behavior and social problems, and attention deficit) [2, 3, 6, 7, 10].

The average age at diagnosis for patients with ADA-SCID prior to the inception of newborn screening in the USA was approximately 4.4 months [8]. Children with ADA-SCID usually die before they reach 2 years old unless they are diagnosed early and effective treatment is initiated [3]. In rare cases, the phenotype is much less severe, with patients presenting after infancy with infections and/or autoimmunity [3]. Early diagnosis with treatment intervention can support an improved prognosis and a more normal life. The overall survival of patients with ADA-SCID was 50% in the 1980s and had increased to 94% by 2010 [11].

Allogeneic hematopoietic stem cell transplant (HCT) or autologous hematopoietic stem cell gene insertion therapy (GT) represent the only definitive therapy options for the immune and hematologic abnormalities associated with ADA-SCID [3, 5]. Enzyme replacement therapy (ERT) is usually the primary treatment for ADA-SCID until patients can receive HCT or GT or when transplant therapy fails to recover immune function to acceptable levels [3, 12] or is not an option [13]. ERT is not curative and must be given regularly for life to maintain a nontoxic metabolic environment [3]. Per consensus guidelines, ERT should be given to all patients with a new diagnosis of ADA-SCID as an immediate stabilizing measure until definitive immune reconstitution with HCT or GT can be achieved [12]. ERT and HCT are currently the only approved therapies available in the USA for the treatment of ADA-SCID.

Until recently, the only ERT available was monomethoxy-polyethylene glycol (PEG)-modified bovine adenosine deaminase (PEG-ADA; pegademase (Adagen®)), which had limitations and theoretical risks [14]. Pegademase is derived from bovine intestines and therefore has the theoretical risk of infection with transmissible spongiform encephalopathies (TSEs), including bovine spongiform encephalopathy, though there is no evidence that this has ever occurred. Additionally, the bovine-sourced enzyme had inherent stability concerns and unwanted proteases that accompanied the product. Inefficient and unreliable production was the major cause of pegademase production uncertainty and ultimately contributed to the desire to replace it with a recombinant product [17].

A major concern with long-term pegademase therapy was the decline in immune reconstitution with prolonged use, with up to 20% of patients becoming treatment-refractory [10, 15]. In patients who had recently begun pegademase therapy, this was partly attributed to the development of neutralizing antibodies to both human and bovine ADA as humoral immunity improves with treatment [3, 10, 16]. Chaffee et al. have described the development of anti-ADA antibody levels in 10 out of 17 patients between 3 and 8 months of treatment; 2 of these patients required pegademase dosing modification (i.e., twice-weekly dosing or withholding pegademase and inducing tolerance with intravenous immunoglobulin and prednisolone prior to restarting pegademase) [16]. Historically, most patients who have remained adherent to therapy on pegademase and survived 6 months after starting treatment had a 90% probability of surviving for the next 12 years [2]. Over time, immunologic integrity tended to fade between 10 and 20 years while on ERT, but for some it lasted longer [12]. Signs of deteriorating immune function included reduction in lymphocyte counts and function and increased susceptibility to infection and other non-infectious complications [12]. The reasons for this decline are not yet known but appeared to not be related to loss of biochemical action of pegademase nor in the development of neutralizing antibodies [12].

In October 2018, the US Food and Drug Administration (FDA) approved recombinant bovine PEG-ADA (elapegademase-lvlr; elapegademase (Revcovi®)) for the treatment of ADA-SCID in pediatric and adult patients [18]. Elapegademase differs from pegademase in several key aspects. Elapegademase is a 113-kDa PEGylated recombinant enzyme produced in *Escherichia coli* utilizing a modified bovine sequence with post-transcriptional removal of the terminal 6 amino acids and capping of cysteine at position 74 by mutating it to serine (Cys74Ser) [14, 19] to reduce oxidative degradation and prolong stability of the protein. Recombinant technology increased the reliability of product yield, lengthened product shelf life, and increased production cost efficiency [17]. PEGylation with a succinimidyl carbonate linker improved product stability with approximately 13 PEG strands [19], in comparison to pegademase PEGylation with approximately 11−17 PEG strands accomplished with more immunogenic succinimidyl succinate linkers [20]. This difference in PEGylation had no significant effect on enzyme activity in vitro [14]. Furthermore, elapegademase has no risk of transmitting TSEs.

Here, we report results from the first study of elapegademase in humans. This phase III trial conducted in the USA evaluated whether elapegademase maintained metabolic detoxification and maintained or improved lymphocyte counts in patients with ADA-SCID currently being treated with pegademase. The trial also assessed the safety of elapegademase through the determination of adverse events (AEs), serious adverse events (SAEs), hospitalizations, and immunogenicity.

## Methods

### Study Design and Treatments

This open-label, multicenter, single-arm, 1-way crossover study (NCT01420627 [21]) in patients with ADA-SCID currently treated with pegademase evaluated the ability of elapegademase to maintain metabolic detoxification and adequate ADA activity with weekly administration. Metabolic detoxification was defined by trough erythrocyte dAXP levels ≤ 0.02 mmol/L (0.02 μmol/mL). Adequate ADA activity was defined by trough plasma ADA activity levels ≥ 15 mmol/h/L (15 μmol/h/mL). The lower limit of quantification (LLOQ) for dAXP was 0.002 mmol/L and for ADA activity was 1.8 mmol/h/L.

This study also assessed safety and tolerability, immunogenicity, and lymphocyte counts. The study was conducted from January 29, 2014, to May 29, 2019, at 6 US centers: National Jewish Health, Denver, CO; Benioff Children’s Hospital, University of California San Francisco, San Francisco, CA; Montefiore Medical Center/Albert Einstein College of Medicine, Bronx, NY; Children’s Hospital Los Angeles, Los Angeles, CA; UBMD/University of Buffalo Jacobs School of Medicine and Biomedical Sciences (formerly the Women and Children’s Hospital of Buffalo), Buffalo, NY; and Penn State Health Hershey Medical Center (formerly the Penn State Milton S. Hershey Medical Center), Hershey, PA.

There were 4 study phases: Screening, pegademase Lead-In Phase, elapegademase Treatment Phase, and elapegademase Maintenance Phase (Supplemental Fig. 1). Screening spanned up to 28 days prior to the start of the pegademase Lead-In Phase to complete screening assessments and meet all eligibility criteria. The pegademase Lead-In Phase was a minimum of 3 weeks and until patients maintained full therapeutic detoxification thresholds for both ADA and dAXP for 2 consecutive weeks, with weekly study visits during this phase. In general, patients who did not meet both therapeutic detoxification criteria prior to transitioning to elapegademase therapy were granted a waiver to enter the elapegademase Treatment Phase on a case-by-case basis. The elapegademase Treatment Phase started after completion of the pegademase Lead-In Phase and spanned a total of 21 weeks, with study visits scheduled every 1 to 3 weeks. The elapegademase Maintenance Phase began after completion of the elapegademase Treatment Phase, with study visits every 3 months, and continued until the commercial availability of elapegademase or early study termination.

### Investigational Products

Both pegademase and elapegademase were supplied by Leadiant Biosciences. Both drugs were manufactured by Exelead, Inc. (formerly known as Sigma-Tau PharmaSource, Inc.), in Indianapolis.

### Pegademase Dose Adjustment

At the start of the pegademase Lead-In Phase, patients on ≥ 2 weekly doses of pegademase had their treatment regimen consolidated into a single weekly dose. Patients who were on once-weekly pegademase dosing were maintained on that regimen at the same dose. Pegademase was administered to all patients at the institutional research site during the pegademase Lead-In Phase. If the patient met the therapeutic thresholds for full therapeutic detoxification (defined as both trough erythrocyte dAXP ≤ 0.02 mmol/L and trough plasma ADA activity ≥ 15 mmol/h/L) for 2 consecutive weeks, they could proceed to the elapegademase Treatment Phase (Supplemental Fig. 1). If the patient did not meet the detoxification criteria, their dosage was adjusted. This process was repeated until the patient met the detoxification criteria. The dose of pegademase was not to be adjusted more than once every 2 weeks to allow the full effect of these changes on trough level of ADA to manifest.

### Elapegademase Dose Selection and Timing

After completion of the pegademase Lead-In Phase, elapegademase was administered weekly. Administration was performed after all procedures and laboratory blood draws for the study visit had been completed. The equivalent dose of elapegademase was calculated as follows:

$$\mathrm{pegademase dose }\left(\frac{\mathrm{U}}{\mathrm{kg}}\right) \times \frac{1\mathrm{ mg elapegademase}}{150\mathrm{ U pegademase}} =\mathrm{elapegademase dose }\left(\frac{\mathrm{mg}}{\mathrm{kg}}\right)$$

The dosage of elapegademase was not adjusted during the study. Patients were maintained on elapegademase therapy until the end of study (EOS).

### Measurement of Treatment Compliance and Home Dosing

Home dosing was permitted beginning at elapegademase Treatment Phase weeks 12, 14, 16, 18, and 20 and during the elapegademase Maintenance Phase until the EOS. Treatment compliance for patients who self-dosed at home was assessed by the site pharmacist, principal investigator, and study coordinator and was verified by clinical research associates during on-site monitoring visits by reviewing diaries and returned vials.

### Study Population

Patients with a diagnosis of ADA-SCID who were clinically stable while receiving pegademase for at least the previous 6 months were enrolled; full eligibility criteria are listed in Supplemental Table 1.

### Efficacy Assessments

The primary endpoint was the proportion of patients who maintained metabolic detoxification during the elapegademase Treatment Phase (weeks 15–21), defined by trough erythrocyte dAXP levels. The secondary endpoints were maintenance of adequate trough ADA activity during the elapegademase Treatment Phase (weeks 15–21) and Maintenance Phase and maintenance of trough dAXP levels during the elapegademase Maintenance Phase. Full therapeutic detoxification was defined as meeting both therapeutic trough dAXP levels and plasma ADA levels. Other secondary efficacy assessments throughout the study included elapegademase effects on total and subset (CD3 + , CD4 + , CD8 + , CD19 + , CD16 + /CD56 +) lymphocyte counts and clinical status (hospitalizations, infections, and overall survival).

### Safety Assessments

Safety was assessed by monitoring AEs and SAEs, number of discontinuations due to AEs, and infections and hospitalizations throughout the study. Safety was also assessed by monitoring for the presence of anti-drug binding, anti-neutralizing, and anti-PEG antibodies throughout the study. Clinical scores were determined using the Lansky Performance Scale (LPS) [22] for patients < 16 years old or the Karnofsky Performance Scale (KPS) [23] for patients ≥ 16 years old.

### Sample Size and Statistical Methods

All study data were summarized descriptively for each patient. All outcomes are reported for the as-treated population, defined as all patients who were enrolled and received at least 1 dose of elapegademase and completed the pegademase Lead-In Phase. Due to notification that Roche was discontinuing production of pegademase and the difficulty of recruiting patients for this study, the FDA agreed to a recruitment of 6 patients. A total of 7 patients were enrolled in the study and received elapegademase. The sample size of 7 patients represents approximately 4% of all patients with ADA-SCID treated with ERT in the USA over the last 2 decades [12]. Inferential statistics were not calculated due to the very small sample size.

## Results

### Patient Demographics

Of 9 patients screened, 7 were enrolled in the study (Table 1). All enrolled patients had a confirmed diagnosis of ADA-SCID and were clinically stable on pegademase. The mean time since patients received their first dose of pegademase was 20.1 years (range, 8–36 years; standard deviation (SD), 8.9 years). Patient 5 was initially enrolled, then withdrawn due to not meeting inclusion criteria for full therapeutic detoxification during the pegademase Lead-In Phase, and later re-enrolled (explained further in the Supplemental Text: Individual Patient Narratives, Patient 5). One patient withdrew consent before the study began. Five of the enrolled patients were diagnosed between birth and 7 months of age, 1 patient was diagnosed at age 3.5 years, and 1 was diagnosed at age 6 years. The mean time since diagnosis for this patient population was 20.4 years (range, 8–37 years; SD, 9.3 years).

**Table 1:** Patient demographics and clinical status

Patient	Sex	Age range, years^a^	Race	Population	Clinical status^b^		Previous therapy
					Start	End	
1	M	< 10	White	As-treated	100	100	-
2	M	19 − 30	Other	As-treated/Completer	40	50	-
3	M	19 − 30	Other	As-treated/Completer	100	100	-
4	M	31 − 40	Black	As-treated/Completer	100	100	Exchange transfusion
5	F	19 − 30	White	As-treated/Completer	100	80	Gene therapy
6	F	10 − 18	White	As-treated/Completer	90	90	-
7	M	10 − 18	White	As-treated/Completer	70	70	-

^a^Age ranges patients fall into were provided in lieu of an exact age to protect patient anonymity

^b^Clinical status at the start and end of the study was determined by measuring LPS (patients < 16 years old) or KPS (patients ≥ 16 years old) *F*, female; *KPS*, Karnofsky Performance Scale [23]; *LPS*, Lansky Performance Scale [22]; *M*, male

Previous or concomitant medications included antibiotics, antifungals, and antivirals; antiseizure medications; inhaled corticosteroids and beta-agonists for asthma; mucolytics and cough suppressants; vitamins; and immunoglobulin (Supplemental Table 2). All patients had a disease-related medical history obtained, such as hearing loss, dermatofibrosarcoma protuberans, blindness, infections, dysphagia causing aspiration with swallowing, and developmental delay (Supplemental Table 2). Previous therapy for ADA-SCID included exchange transfusion resulting in partial immune reconstitution and failed GT (Table 1). A full list of previous therapies for ADA-SCID, concomitant medications, and patient medical history can be found in Supplemental Table 2.

Pegademase dosing was adjusted during the Lead-In Phase for patients 3, 4, and 7 until they reached full therapeutic detoxification (Table 2). The remaining 4 patients did not require dose adjustment to reach full therapeutic detoxification. The mean (SD) duration of pegademase therapy was 6.3 (3.45) weeks.

**Table 2:** Pegademase dose adjustment and elapegademase dosage

	Screening	Lead-In Phase		Treatment and Maintenance Phase		
Patient	Pegademase regimen, no. of injections/week	Pegademase, U/kg^a^		Elapegademase, mg/kg^b^	Treatment duration, years	Elapegademase self-dosing^c^
		Start	End			
1^d^	2	43.9	43.4	0.28	0.1	No
2	2	28.2	27.9	0.19	4.2	Yes
3	1	29.6	36.1	0.23	2.4	No
4	2	7.7	30.0	0.20	4.2	No
5	1	31.3	32.2	0.21	2.4	No
6	2	42.9	44.1	0.29	2.1	Yes
7	3	21.6	26.2	0.17	1.4	Yes

^a^Total dose for the week

^b^Elapegademase dosage was based on pegademase dosage, using a conversion factor for enzyme equivalent activity

^c^Elapegademase self-dosing at home was allowed during the Treatment Phase from weeks 12, 14, 16, 18, and 20 and during the Maintenance Phase until the end of the study

^d^Patient 1 received 2 doses of elapegademase and withdrew due to a treatment-emergent adverse effect

1 mg elapegademase = 150 U pegademase

Seven patients received at least 2 doses of elapegademase (Table 2). Patient 1 withdrew from the study after 2 doses of elapegademase due to an AE of severe injection-site pain from an early formulation of the study drug containing EDTA. EDTA was subsequently removed from the formulation (Supplemental Text: Individual Patient Narratives, Patient 1). This patient was included in the pegademase efficacy, immunogenicity, and safety analyses but not in the elapegademase efficacy analysis. The remaining 6 patients completed the study and received elapegademase formulated without EDTA (also known as elapegademase-lvlr but referred to as elapegademase herein). The 6 remaining patients completed 71–216 weeks of elapegademase therapy, with 3 of these completing ≥ 212 weeks. The mean (SD) duration of elapegademase treatment for the entire treated population was 135.7 (83.53) weeks. Three completer patients participated in self-dosing at home during elapegademase therapy (Table 2).

### Metabolic Detoxification

**Fig. 1:**
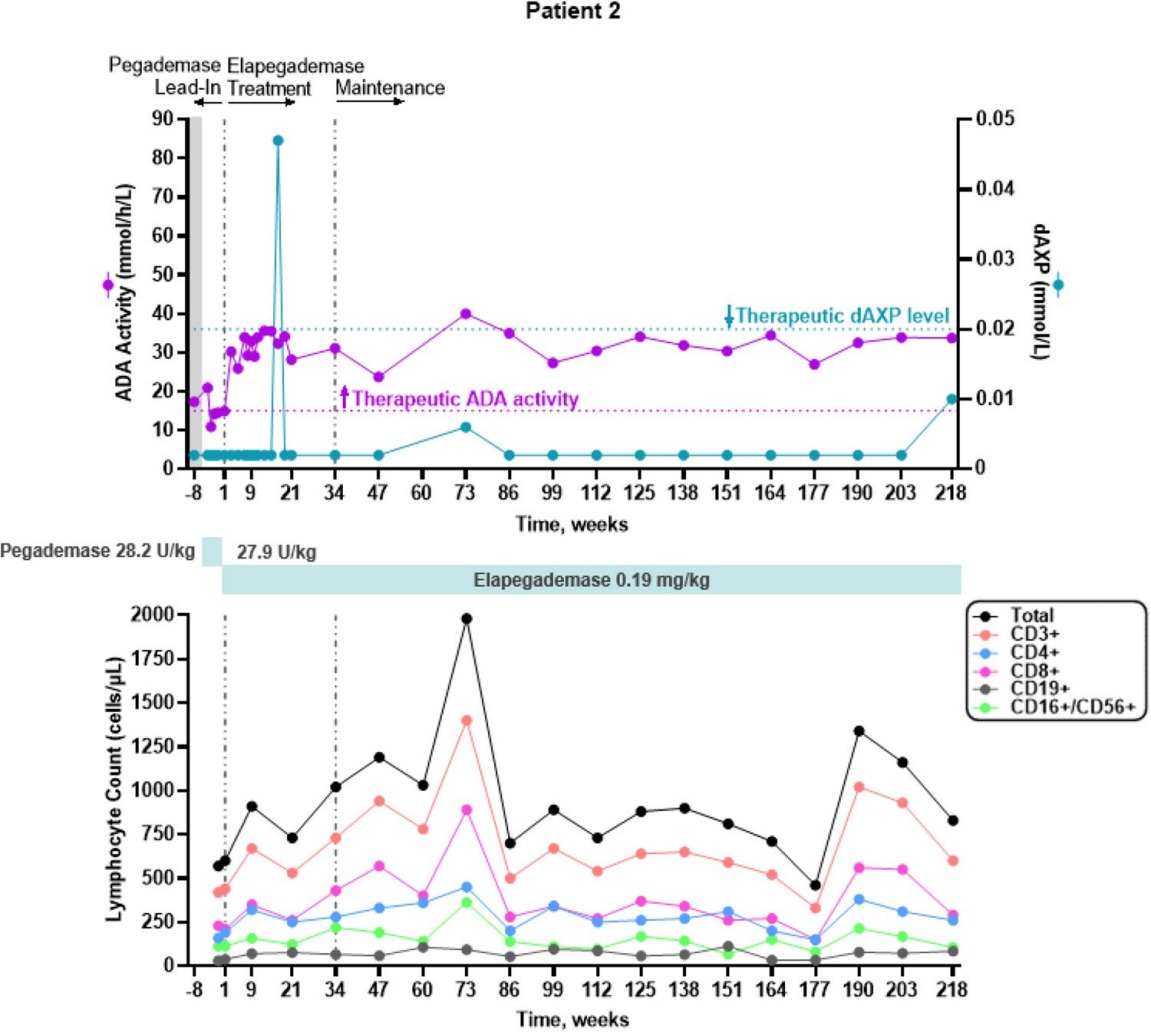
Patient 2: metabolic detoxification and lymphocyte levels throughout the study. Values obtained at Screening are indicated by a shaded gray box. Cut-off values for meeting full metabolic detoxification are indicated in the top panel by the horizontal dashed lines, colored to match the corresponding data set. Screening values were obtained while patients were on pegademase and may not be trough values

**Fig. 2:**
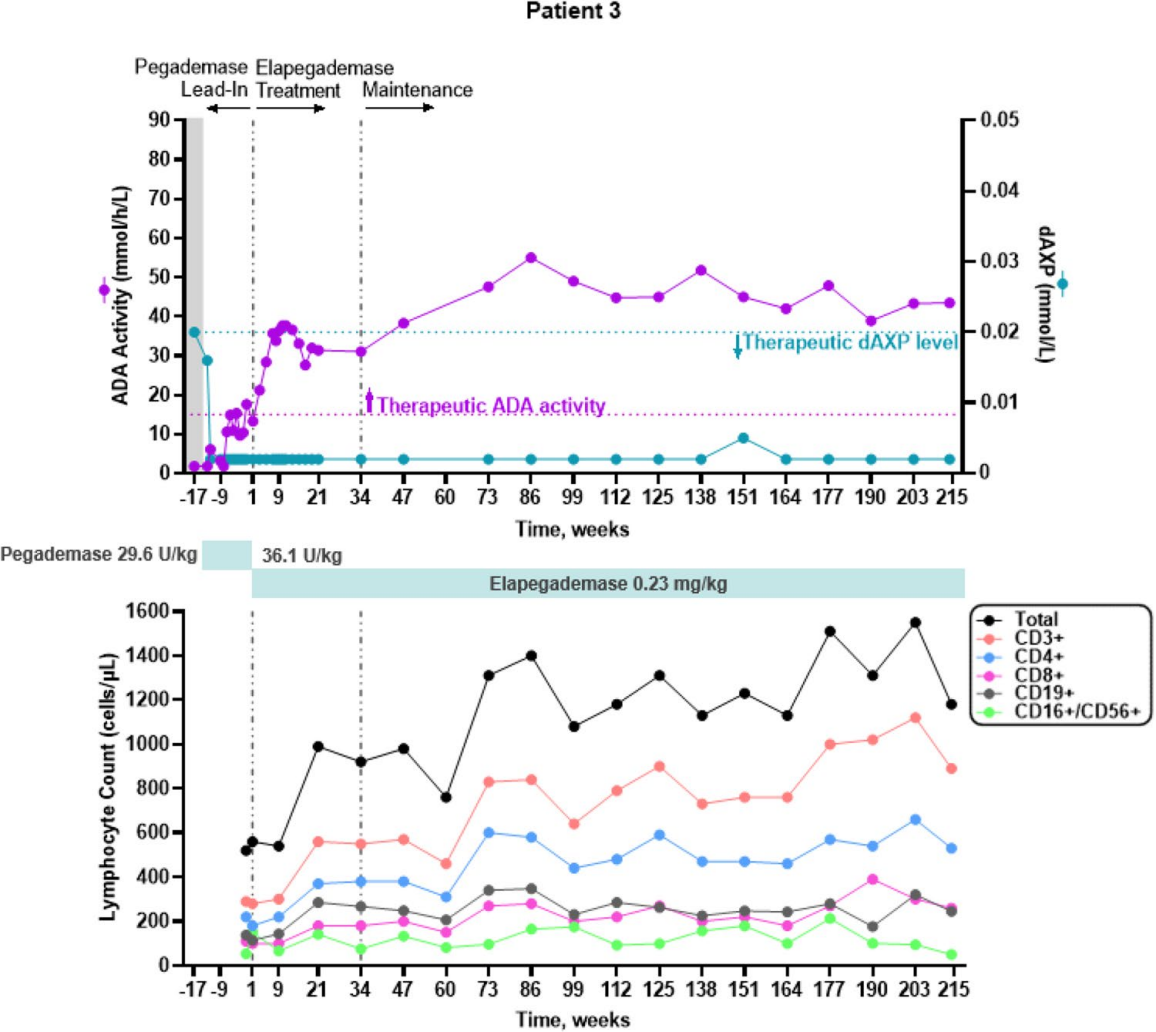
Patient 3: metabolic detoxification and lymphocyte levels throughout the study. Values obtained at Screening are indicated by a shaded gray box. Cut-off values for meeting full metabolic detoxification are indicated in the top panel by the horizontal dashed lines, colored to match the corresponding data set. Screening values were taken while patients were on pegademase and may not be trough values

**Fig. 3:**
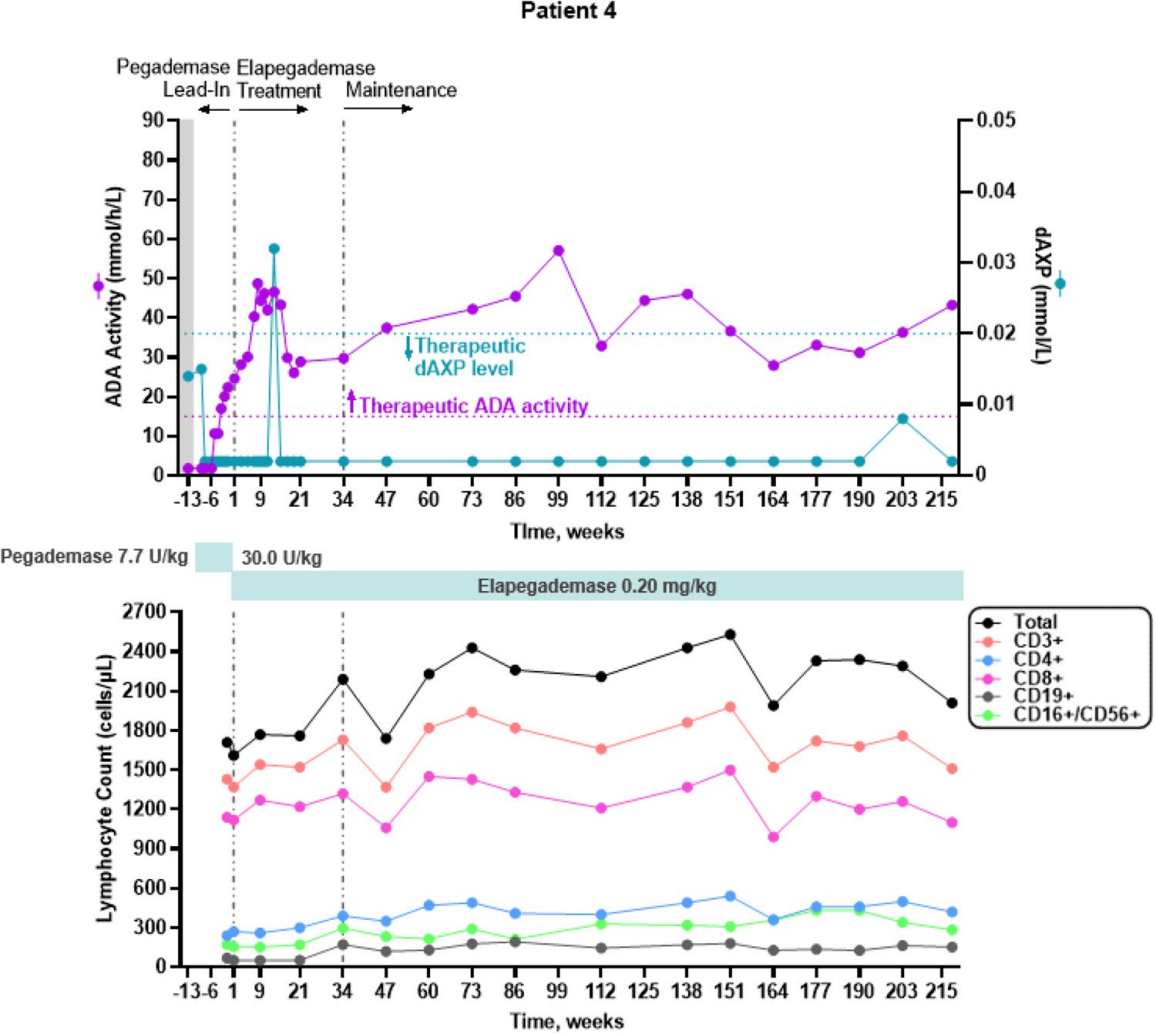
Patient 4: metabolic detoxification and lymphocyte levels throughout the study. Values obtained at Screening are indicated by a box shaded gray. Cut-off values for meeting full metabolic detoxification are indicated in the top panel by the horizontal dashed lines, colored to match the corresponding data set. Screening values were taken while patients were on pegademase and may not be trough values

**Fig. 4:**
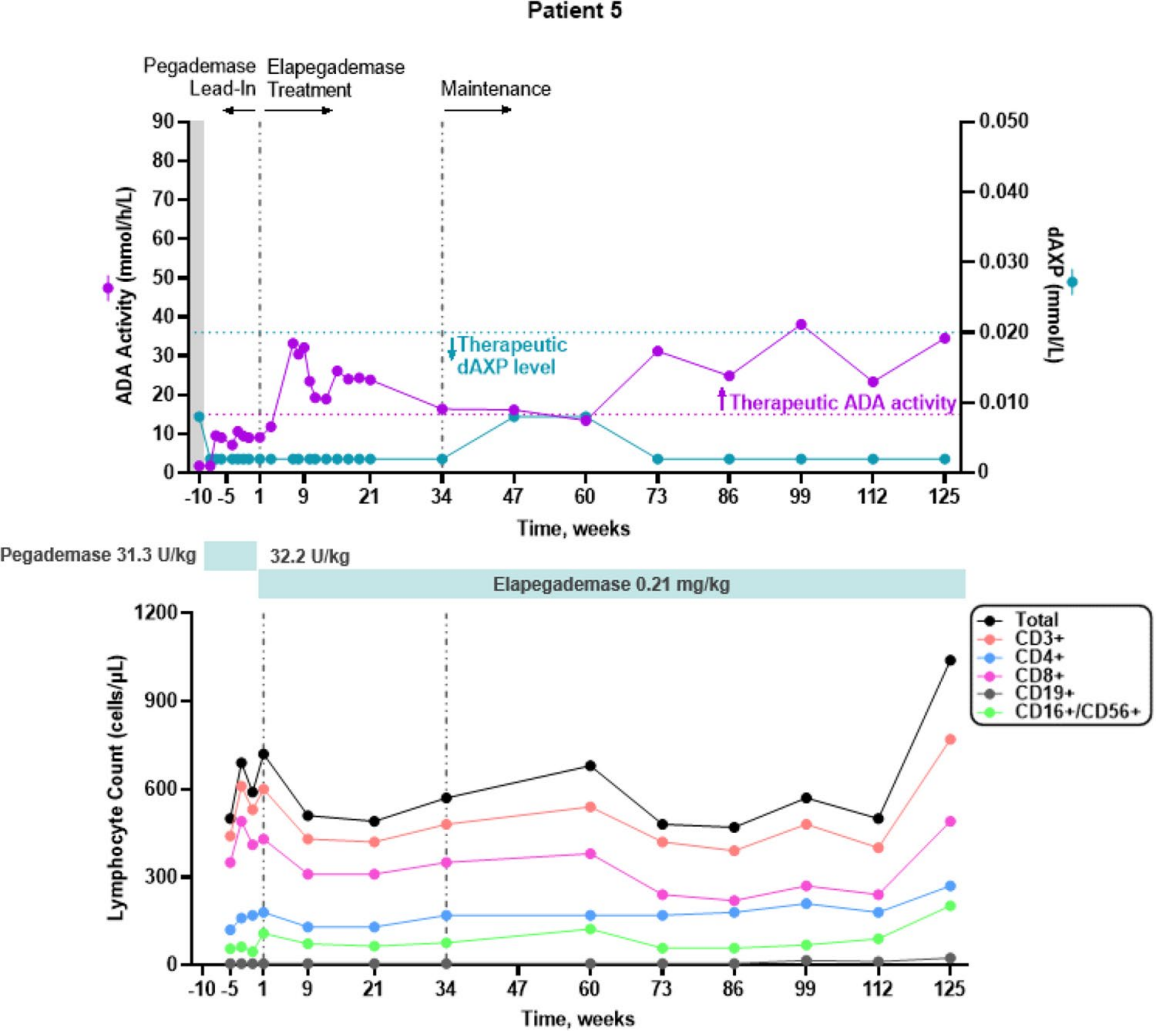
Patient 5: metabolic detoxification and lymphocyte levels throughout the study. Values obtained at Screening are indicated by a box shaded gray. Cut-off values for meeting full metabolic detoxification are indicated in the top panel by the horizontal dashed lines, colored to match the corresponding data set. Screening values were taken while patients were on pegademase and may not be trough values

**Fig. 5:**
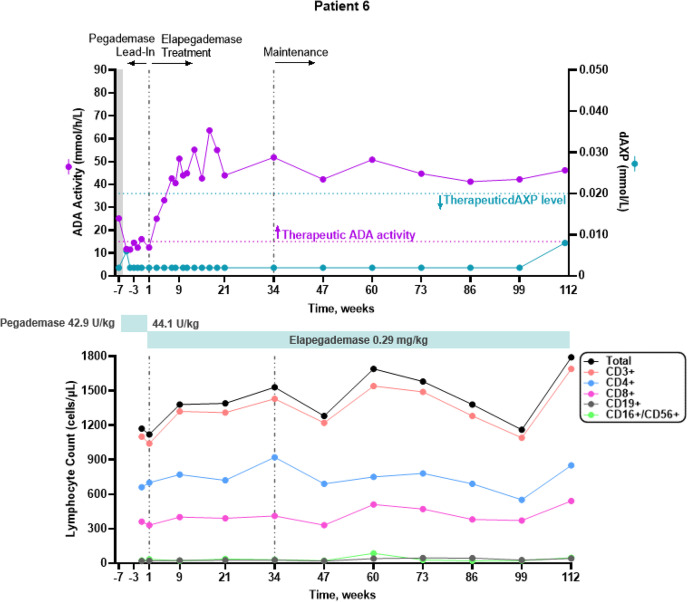
Patient 6: metabolic detoxification and lymphocyte levels throughout the study. Values obtained at Screening are indicated by a box shaded gray. Cut-off values for meeting full metabolic detoxification are indicated in the top panel by the horizontal dashed lines, colored to match the corresponding data set. Screening values were taken while patients were on pegademase and may not be trough values

**Fig. 6:**
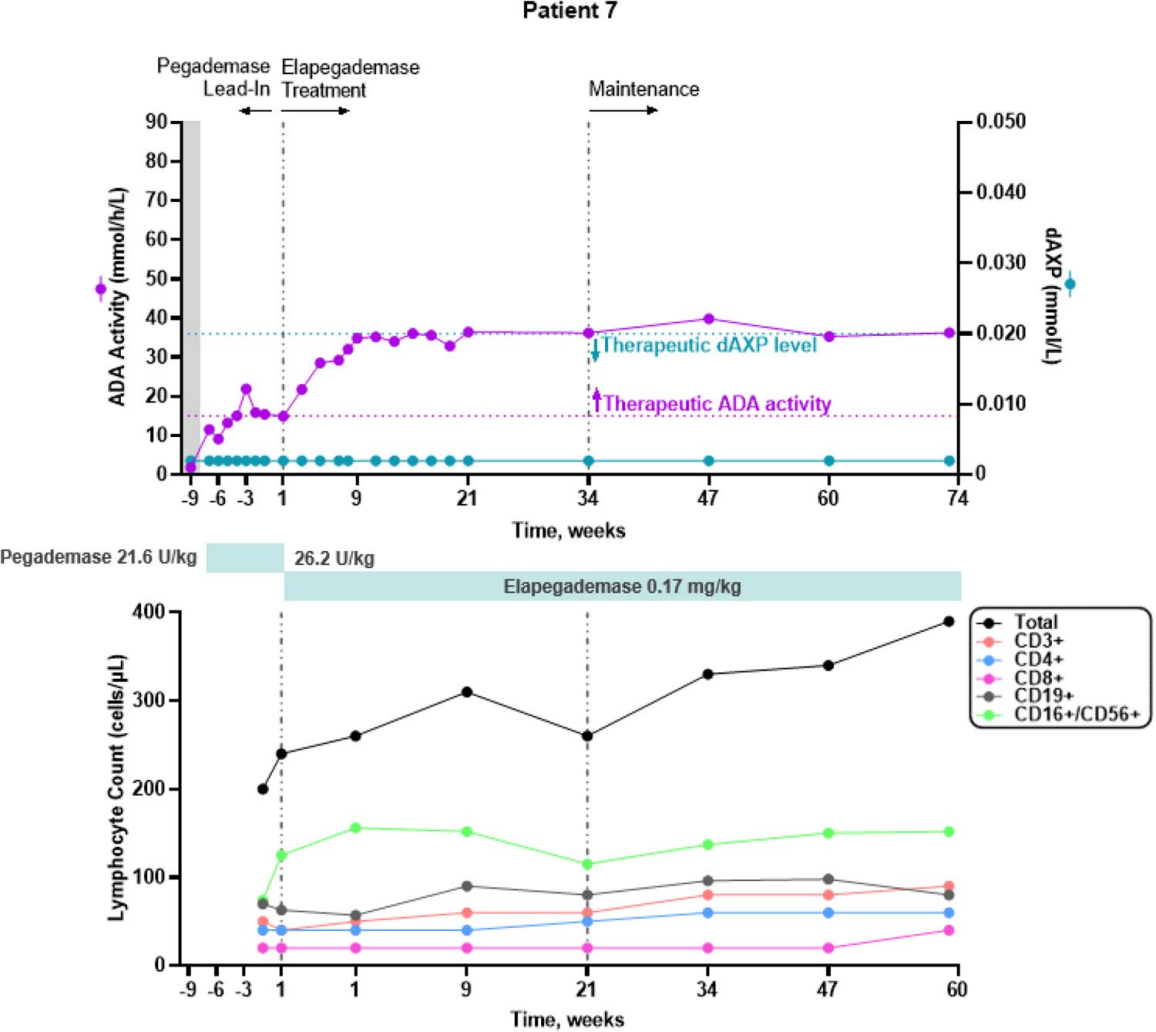
Patient 7: metabolic detoxification and lymphocyte levels throughout the study. Values obtained at Screening are indicated by a box shaded gray. Cut-off values for meeting full metabolic detoxification are indicated in the top panel by the horizontal dashed lines, colored to match the corresponding data set. Screening values were taken while patients were on pegademase and may not be trough values

All 7 patients were considered metabolically detoxified at Screening based on their trough erythrocyte dAXP levels (Figs. 1, 2, 3, 4, 5, and 6, top panels). At Screening, while the patients were on pegademase, 4 of the 7 patients had trough plasma ADA activity levels < 15 mmol/h/L and were therefore considered not to have therapeutic ADA activity levels (Figs. 1, 2, 3, 4, 5, and 6, top panels).

At the end of the pegademase Lead-In Phase, all 7 patients had trough erythrocyte dAXP levels ≤ 0.02 mmol/L (Figs. 1, 2, 3, 4, 5, and 6, top panels), though Patients 2, 3, 5, and 6 did not meet the criteria for elapegademase transition based on their trough ADA activity levels. Patients 2, 3, 5, and 6 had trough ADA activity levels below the therapeutic cut-off at 14.2, 10.4, 9.5, and 12.4 mmol/h/L, respectively. Since dAXP levels were ≤ 0.02 mmol/L and ADA activity levels were close to the protocol required level, the patients were considered stable and detoxified and allowed to continue in the study.

Trough plasma ADA activity values increased during the elapegademase Treatment Phase and were consistently > 15 mmol/h/L for all patients after week 3 (Figs. 1, 2, 3, 4, 5, and 6, top panels). Mean (SD) ADA activity at the end of study (elapegademase, 39.6 [5.3] mmol/h/L) was approximately twice the levels of pegademase at baseline (17.1 [3.5] mmol/h/L). Of the 6 patients who received elapegademase through week 21, 5 met the predefined criterion for maintenance of metabolic detoxification during weeks 15–21. All patients had dAXP levels below 0.02 mmol/L at all time points, except for Patient 2, who had a dAXP level of 0.047 mmol/L at week 17; however, this patient had dAXP levels below 0.02 mmol/L at all other time points during elapegademase therapy up to the EOS at 218 weeks (Fig. 1, top panel). Patient 1, who withdrew due to severe injection-site pain/discomfort, was fully detoxified at early discontinuation (data not shown).

With 1 transient exception, all patients maintained full therapeutic detoxification throughout the elapegademase Maintenance Phase until the EOS. Patient 5 had reduced ADA activity below the therapeutic threshold at the week 60 visit that improved above the therapeutic threshold at the subsequent visit and was stable thereafter (Fig. 4, top panel). All 6 patients who completed the study were metabolically detoxified at EOS (Figs. 1, 2, 3, 4, 5, and 6, top panels).

### Lymphocyte Reconstitution

Lymphocyte subset counts were measured throughout the study and included CD3 + , CD4 + , CD8 + , CD19 + , and CD16 + /CD56 + (Figs. 1, 2, 3, 4, 5, and 6, bottom panels). Total lymphocyte counts varied between patients and increased or were maintained during the study following elapegademase therapy in comparison with pegademase therapy; all patients had increases in total lymphocyte counts between 1.2- and 2.1-fold at EOS compared with baseline (i.e., the end of the pegademase Lead-In Phase).

While all patients experienced an increase in trough plasma ADA activity levels, there was an increase in some lymphocyte subsets in all 6 completer patients (Figs. 1, 2, 3, 4, 5, and 6, top versus bottom panels). All patients had increased CD3 + (range, 1.1–3.2-fold), CD4 + (range, 1.2–2.9-fold), and CD19 + (range, 1.3–4.8-fold) counts at EOS compared with counts obtained at baseline. CD8 + counts were higher at EOS compared with baseline in 5 of 6 patients (fold-change range, 1.0–2.6), and CD16 + /56 + counts were higher at EOS compared with baseline in 4 of 6 patients (fold-change range, 0.3–1.9).

### Immunogenicity

**Fig. 7:**
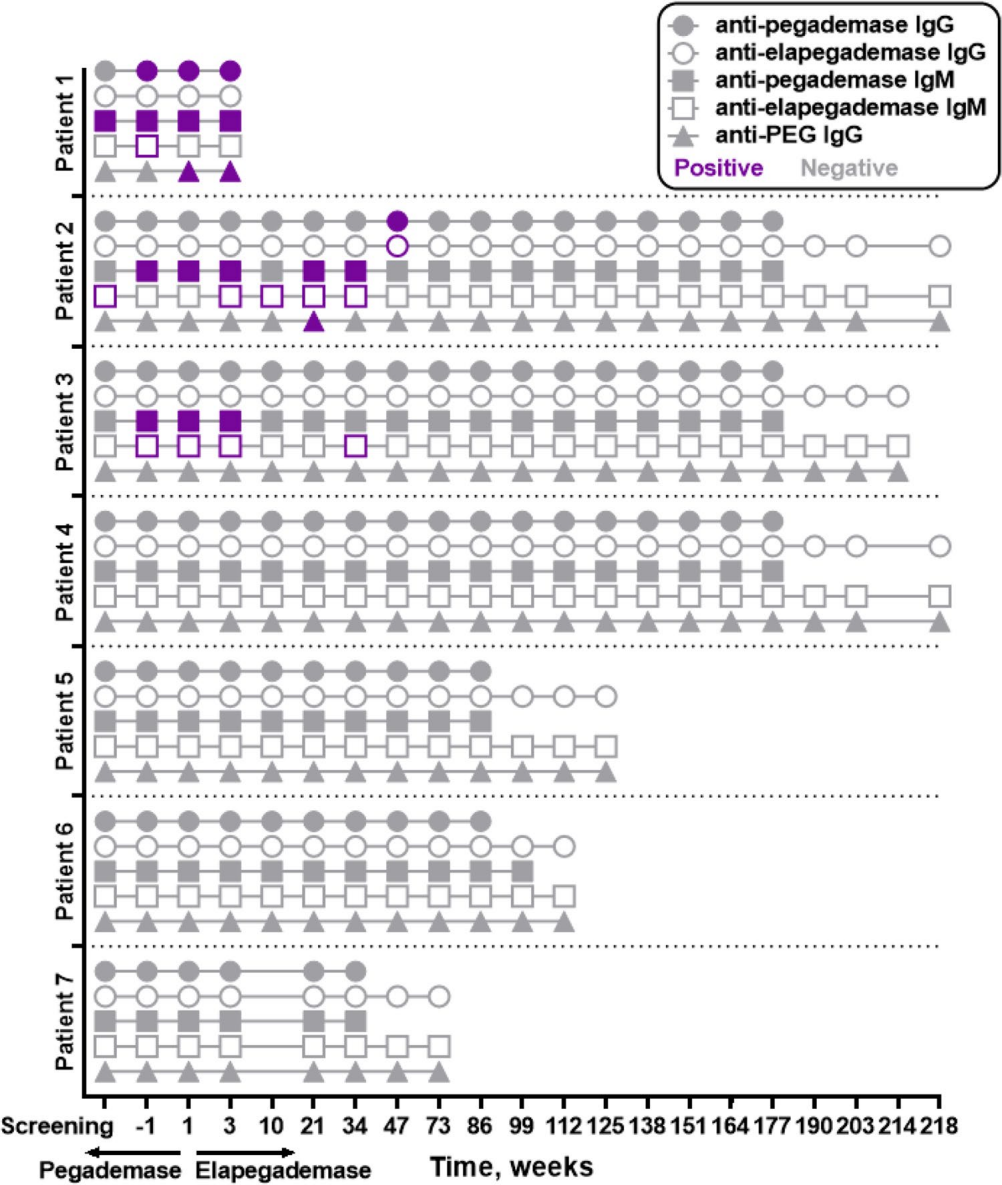
Presence of anti-drug antibodies during elapegademase therapy. Based on test results for first tier (“immunoggenicity” in source listing) and second tier (“immuno-2” in source listing; performed only if first-tier result was positive)

All patients were negative for anti-drug neutralizing antibodies throughout the study (data not shown). Three of the 7 treated patients (Patients 1, 2, and 3) had transient, non-neutralizing anti-drug immunoglobulin G (IgG) and/or immunoglobulin M (IgM) antibodies against pegademase and elapegademase at or before 47 weeks at ≥ 1 visit during both the pegademase Lead-In Phase and elapegademase therapy (Fig. 7).

Two of these patients also had transient anti-PEG antibodies at 1 visit during elapegademase therapy. The anti-PEG antibody levels for Patient 1 did not rise; their titer was the same at withdrawal compared with when they started elapegademase. The only other patient positive for anti-PEG antibodies had transient titers for one study visit. No patient who had either anti-drug IgG or IgM antibodies had commensurate alterations of therapeutic endpoints (i.e., no related increase in dAXP or reduction in ADA activity).

### Safety

A total of 10 AEs were reported during the pegademase Lead-In Phase (mean, 6.2 weeks exposure per patient; Table 3). All were mild or moderate. Only 1 of these AEs (Patient 1, abnormally low hemoglobin) was assessed as possibly related to study medication. There were no changes in study medication dosing due to AEs during the pegademase Lead-In Phase, and none were assessed as serious.

**Table 3:** Occurrence of adverse events during the study

	Pegademase *N* = 7	Elapegademase *N* = 7
Treatment-emergent event, *n* (%)	6 (85.7)	7 (100.0)
Maximum severity of AE, *n* (%)		
Mild	4 (57.1)	1 (14.3)
Moderate	2 (28.6)	3 (42.9)
Severe	0 (0.0)	3 (42.9)
Treatment-related event, *n* (%)	1 (14.3)^a^	2 (28.6)^b^
Maximum severity of treatment-related AE, *n* (%)		
Mild	1 (14.3)	1 (14.3)
Moderate	0 (0.0)	0 (0.0)
Severe	0 (0.0)	1 (14.3)
Patients with any SAE, *n* (%)	0 (0.0)	4 (57.1)
Patients discontinued treatment due to AE, *n* (%)	0 (0.0)	1 (14.3)^c^
Nonserious AE, *n* (%)	6 (85.7)	7 (100.0)

^a^Patient 1 had an abnormally low hemoglobulin, possibly related to study medication

^b^Patient 5 had mild injection-site discomfort/sensitivity and Patient 1 had 2 incidents of injection-site pain (1 moderate and 1 severe) that led to study withdrawal

^c^Injection-site pain (severe)

*AE*, adverse event; *SAE,* serious adverse event

There were 131 AEs reported during elapegademase therapy (mean, 135.7 weeks of exposure per patient), and all patients experienced treatment-emergent AEs (Table 3). Three patients each had vomiting and cough, and 2 patients each had productive cough, upper respiratory infection, nausea, noncardiac chest pain, oropharyngeal pain, and/or pyrexia. Three patients reported 4 AEs assessed as severe (injection-site pain, tooth abscess, tooth extraction, and superior vena cava stenosis). Four patients reported 8 AEs assessed as serious (2 events of injection-site pain in 1 patient, dehydration and vestibular migraine in 1 patient, tooth abscess and tooth extraction in 1 patient, and respiratory tract infection and hemoptysis in 1 patient). With the exception of injection-site pain, the severe and serious AEs were not related to elapegademase therapy; rather, they were related to disease state. In total, 23 AEs assessed as related to elapegademase therapy were reported for 2 patients. All were categorized as injection-site pain/discomfort, including a serious AE that led 1 patient to withdraw from the study due to the EDTA-containing drug product (see Supplemental Text: Individual Patient Narratives, Patient 1). All other AEs were reported for only 1 patient. With the exception of the serious injection-site pain that led to study withdrawal in 1 patient, AEs did not lead to changes in dosing.

### Infections and Hospitalizations

A range of 1 to 5 mild or moderate infections were reported per patient during the study. Five patients had 26 infections (Table 4), and all but 2 were resolved at EOS. Patient 7 had a case of epidermodysplasia verruciformis that began during the pegademase Lead-In Phase, was nonserious, and was ongoing at EOS. Patient 5 had a respiratory tract infection that began during week 84 of elapegademase treatment and was ongoing at EOS. These incidents are described in further detail in the Supplemental Text: Individual Patient Narratives.

**Table 4:** Summary of infections and hospitalizations

	Elapegademase *N* = 7
Patients with infection, *n* (%)	5 (71.4)
Total number of infections, *n*	26
Patients with hospitalization, *n* (%)	3 (42.9)
Total number of hospitalizations, *n* (%)	
1	1 (14.3)
2	2 (28.6)
3	0 (0.0)
Duration of hospitalization, days^a^	
*n*	4
Mean	5.0
Standard deviation	2.16
Median	5.5
Minimum	2
Maximum	7
Reason for hospitalization, *n*^b^	
Dehydration	1
Hemoptysis	1
Respiratory tract infection	1
Tooth abscess	1
Vestibular migraine	1

^a^Duration of hospitalization = (end date of hospitalization – start date) + 1

^b^Patients can be admitted more than once, so “*n*” can be larger than the number of patients, and row totals can sum to more than the number of patients

Three patients were hospitalized with serious AEs deemed unrelated or unlikely to be related to the study drug (Table 4), and they were resolved before the EOS (see Supplemental Text: Individual Patient Narratives for additional details). Patient 2 was hospitalized for 1 day to treat dehydration and weakness due to vestibular migraines. Patient 4 was hospitalized for 4 days due to a tooth abscess. Patient 5 was hospitalized twice: first for respiratory tract infection and later for hemoptysis. The mean (range) duration of hospitalization was 5 (2–7) days. Overall, no substantial changes in clinical status were observed at EOS compared to Screening (Table 1).

## Discussion

Upon entering the elapegademase Treatment Phase, all patients had an increase in ADA activity that was maintained throughout the study, indicating that elapegademase is effective at sustaining therapeutic ADA levels. Two patients had transient increases above the 0.02 mmol/L threshold in trough erythrocyte dAXP at 1 time point during the elapegademase Treatment Phase. Furthermore, 1 patient had decreased trough plasma ADA activity levels below the threshold that spanned 1 visit (13.4 mmol/h/L) during the elapegademase Maintenance Phase. Despite these transient ADA decreases during elapegademase therapy, trough erythrocyte dAXP levels were ≤ 0.02 mmol/L, and trough plasma ADA activity levels were ≥ 15 mmol/h/L at almost all time points. These data indicate that adherence to a weekly elapegademase therapy regimen was effective at maintaining full therapeutic detoxification in patients with ADA-SCID.

Six patients had significant lymphopenia at screening, including abnormally low total lymphocyte and CD3 + counts. Importantly, total lymphocyte counts increased or were maintained throughout the study until EOS during elapegademase therapy compared with pegademase therapy. At EOS, all patients had higher total lymphocyte counts than at baseline, and 2 patients had CD3 + lymphocyte counts improve to ~ 1500 cells/µL over the course of the study, indicating that elapegademase therapy provided these patients with improvements in their total lymphocyte counts. However, the improvement in lymphocyte counts did not reach normalization in all patients.

Long-term pegademase therapy is associated with the development of anti-pegademase antibodies in up to 80% of patients [10]. In about 10% of treated patients, neutralizing antibodies are produced and lead to enhanced clearance of pegademase, a subsequent increase in dAXP levels, and a decline in immune function [10]. In this study, most immunogenicity tests were negative for antibodies (anti-pegademase, anti-elapegademase, or anti-PEG antibodies), and no patient had neutralizing antibodies against the study drug or PEG. Three patients had transient positive results for anti-elapegademase antibodies that were non-neutralizing and occurred early during the study (pegademase Lead-In Phase) likely due to the presence of cross-reactive anti-pegademase antibodies in these patients. No patient developed anti-elapegademase antibodies after week 47. The presence of anti-drug antibodies did not affect any efficacy or safety outcomes. No apparent pattern was observed with the presence or absence of anti-drug antibodies against pegademase and elapegademase, but the small sample size was likely insufficient to detect such a pattern. Longer follow-up in a larger population will be necessary to assess this issue.

Due to the ultrarare nature of ADA-SCID, clinical experience with pegademase is limited. Hemolytic anemia, thrombocytopenia, lymphomas, and injection-site reactions were some of the voluntarily reported pegademase AEs [24]. The number of treatment-emergent AEs in this study was similar between pegademase and elapegademase therapy, though the duration of pegademase therapy was ≤ 12 weeks compared with the much longer duration of elapegademase therapy of 71–216 weeks. Most AEs that emerged during elapegademase therapy were nonserious, mild, and unrelated to study treatment, and most were resolved by EOS. The most common AEs were cough, vomiting, and injection-site pain/discomfort. Patient 1 experienced injection-site pain, due to EDTA in the study drug formulation, which led to the patient’s withdrawal from the study. Investigations of this patient’s reaction led to removal of EDTA from the formulation for subsequent use during this trial and the FDA-approved drug formulation does not contain EDTA. It should be emphasized that Patient 1 received a formulation other than elapegademase-lvlr due to the presence of EDTA. In this study, no patient experienced hemolytic anemia, thrombocytopenia, or lymphoma, and all patients were clinically stable.

Reducing dosing frequency has been shown to improve quality of life for patients [25, 26]. While weekly pegademase dosing was recommended in the product insert [24], patients with reduced ADA activity levels affecting their clinical status required dosing modifications to achieve a therapeutic effect. A previous report by Chafee et al. described 1 such patient who responded to twice-weekly injections [16]. The product insert for pegademase stated that 1 of 12 patients show enhanced clearance of plasma ADA activity after 5 months of therapy at the recommended dose, requiring these patients to be treated twice weekly at an increased dose for several months before returning to weekly administration [24]. In this study, 5 patients were on 2–3 administrations of pegademase weekly in an attempt to improve lymphocyte counts. Upon study initiation, patients were consolidated to pegademase therapy once weekly and maintained a once-weekly dosing schedule with no elapegademase dose adjustments upon switchover. Except for one patient at one visit, all completer patients maintained full therapeutic detoxification during elapegademase therapy with no increased incidence of AEs, no indication of clinical deterioration, and no increase in the development of anti-drug antibodies for up to 4.2 years of therapy.

A major strength of this study is the frequent laboratory and hematological measures, allowing for comparison between pegademase and elapegademase therapy outcomes. Limitations of the study include the small patient population, lack of assessment of treatment-naïve patients, and limited immune function analysis. Based on these data and data from a clinical trial with a Japanese cohort [27], the FDA approved elapegademase for the treatment of adult and pediatric patients with ADA-SCID in October 2018 [18].

## Conclusion

Elapegademase provided therapeutic trough plasma ADA activity and safely maintained metabolic detoxification in patients with ADA-SCID who were previously on pegademase therapy. No new safety concerns related to elapegademase therapy were reported in this patient population. Based on this study, patients who had previously been on pegademase therapy and/or exchange transfusion or failed GT could experience improvements in therapeutic ADA activity levels and metabolic detoxification, subsequent lymphocyte maintenance or reconstitution after switching to elapegademase therapy.

A plain language summary of this study is available as supplemental material (Supplemental Text: Elapegademase treatment for people with ADA-SCID).

## Supplementary Information

Below is the link to the electronic supplementary material.

Supplementary file1 (DOCX 1961 KB)

## Data Availability

At this time, Chiesi will approve or deny data requests from external parties on a case-by-case basis. Chiesi reserves the right to deny requests for any and all legally appropriate reasons. Data requests that risk sharing participant-level data or proprietary information will not be approved.
