# Correction to: PEGylated Recombinant Adenosine Deaminase Maintains Detoxification and Lymphocyte Counts in Patients with ADA-SCID

**DOI:** 10.1007/s10875-023-01531-6

**Published:** 2023-06-09

**Authors:** Morna J. Dorsey, Arye Rubinstein, Heather Lehman, Tracy Fausnight, Joseph M. Wiley, Elie Haddad

**Affiliations:** 1 Pediatric Immunology and Allergy Center, University of California San Francisco Medical School, San Francisco, CA USA; 2 Department of Pediatrics, Montefiore Medical Center, Albert Einstein College of Medicine, Bronx, NY USA; 3 Department of Pediatrics, University of Buffalo Jacobs School of Medicine and Biomedical Sciences, Buffalo, NY USA; 4 Department of Pediatrics, Penn State Health Hershey Medical Center, Hershey, PA USA; 5 Medical Affairs, Leadiant Biosciences, Inc, Gaithersburg, MD USA; 6 Department of Pediatrics, University of Montreal, Montreal, QC Canada

**Correction to** **:** **Journal of Clinical Immunology**

**https://doi.org/10.1007/s10875-022-01426-y**

The original version of this article contained an error in Table 4 under “total number of hospitalizations”. The corresponding information in the text was correct. The corrected table is presented below:

**Table 4:** Summary of infections and hospitalizations

	Elapegademase *N* = 7
Patients with infection, *n* (%)	5 (71.4)
Total number of infections, *n*	26
Patients with hospitalization, *n* (%)	3 (42.9)
Total number of hospitalizations, *n* (%)	
1	2 (28.6)
2	1 (14.3)
3	0 (0.0)
Duration of hospitalization, days	
* n*	4
Mean	5.0
SD	2.16
Median	5.5
Min	2
Max	7
Reason for hospitalization, n^a^	
Dehydration	1
Hemoptysis	1
Respiratory tract infection	1
Tooth abscess	1
Vestibular migraine	1

Duration of hospitalization = (end date of hospitalization – start date) + 1.

^a^Patients can be admitted more than once so “n” can be larger than the number of patients, and row totals can sum to more than the number of patients

*Max*, maximum, *Min*, minimum, *SD*, standard deviation.

