# Supplementary material for: PEGylated Recombinant Adenosine Deaminase Maintains Detoxification and Lymphocyte Counts in Patients with ADA-SCID
Source: J Clin Immunol. 2023 Feb 25;43(5):951–64. doi: 10.1007/s10875-022-01426-y (PMC10276086; doi:10.1007/s10875-022-01426-y)

# Supplementary Information

## **Supplemental Figure 1**

Study design. The 4 study phases and their durations are indicated. Relevant inclusion criteria and the primary and secondary endpoints are listed below the corresponding study phases, where applicable.


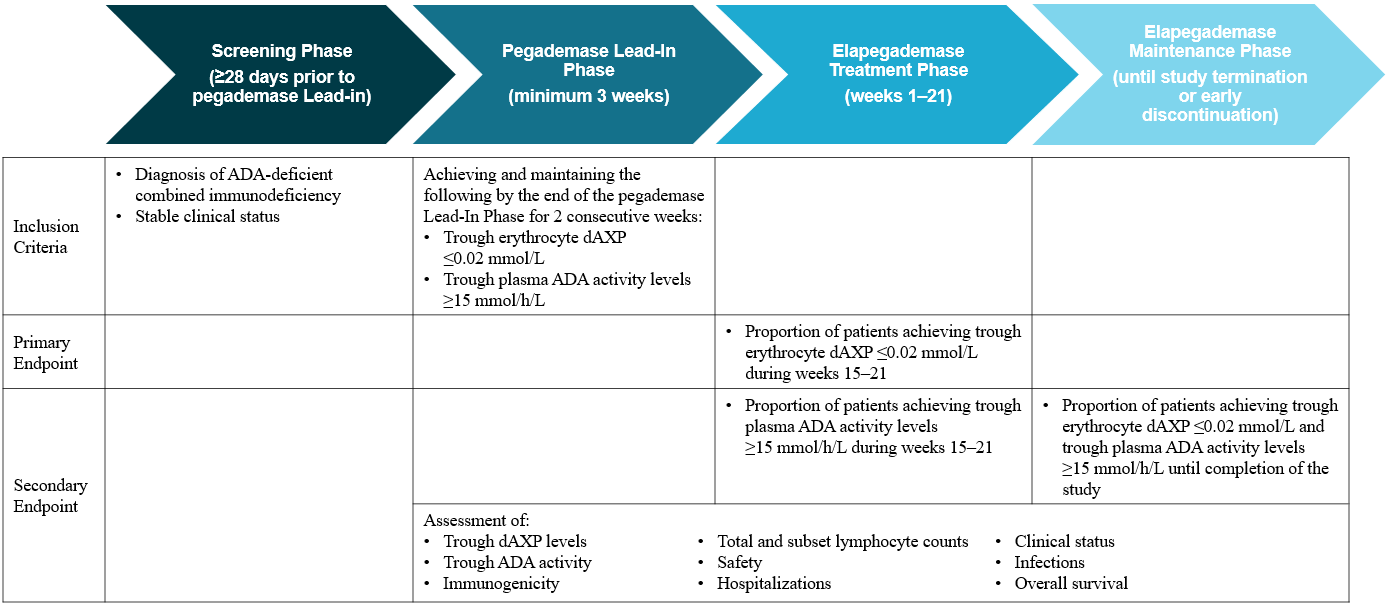


## **Supplemental Table 1**

Summary of key inclusion and exclusion criteria

| Inclusion criteria | - Diagnosis of ADA-deficient combined immunodeficiency - Stable clinical status while receiving therapy with pegademase - Patients previously receiving GT or undergoing HCT who still required pegademase treatment were eligible   - The dose of pegademase was to be stable for at least 6 months prior to study entry - Have both of the following during the pegademase Lead-in Phase of the study prior to elapegademase transition:   - Trough plasma ADA activity ≥15 mmol//h/L while receiving pegademase   - Total erythrocyte dAXP ≤0.02 mmol/L from a trough blood sample - Patients or parent/guardian were to be capable of understanding the protocol requirements and risks and providing written informed assent/consent |
| --- | --- |
| Exclusion criteria | - Autoimmunity requiring immunosuppressive treatment - Patients with detectable neutralizing anti-pegademase antibodies at Screening evaluation - Severe thrombocytopenia (platelet count <50×10^9^/L) - Current participation in other therapeutic protocols for ADA-deficient combined immunodeficiency - Current or prior participation in another clinical study with an investigational agent and/or use of an investigational drug in the 30 days before study entry - Known planned participation in a gene therapy study for the planned duration of this study - Any condition that, in the opinion of the PI, made the patient unsuitable for the study - Inability or unwillingness to administer pegademase or elapegademase on a one time per week regimen - Inability to comply with the study protocol - Female patients who were pregnant, lactating, breast-feeding, or of childbearing potential who were not using an FDA-approved birth control method |

*ADA*, adenosine deaminase activity; *dAXP*, intracellular adenosine and deoxyadenosine nucleotides; *FDA*, U.S. Federal Drug Administration; *GT,* gene therapy; *HCT*, hematopoietic stem cell transplant; *PI,* principal investigator

## **Supplemental Table 2**

Concomitant medications*^a^* and medical history

| **Patient** | **Age at Diagnosis (years)** | **Prior Therapies**  **for ADA-SCID** | **Concomitant Medications (Pegademase)** | **Concomitant Medications (Elapegademase)** | **Medical History** |
| --- | --- | --- | --- | --- | --- |
| 1 | 0.4 | - Pegademase | - Trimethoprim/sulfamethoxazole - Immunoglobulin human normal - Amoxicillin - Paracetamol/acetaminophen | - Trimethoprim/sulfamethoxazole - Immunoglobulin human normal - Amoxicillin - Paracetamol/acetaminophen | - Hearing loss both ears - Ear scar on left Tympanic membrane - Toe walker - Fever |
| 2 | 0.3 | - Pegademase | - Immunoglobulin human normal - Carbamazepine - Trimethoprim/sulfamethoxazole - Risperidone - Macrogol 3350 - Ergocalciferol - Cetirizine hydrochloride - Salbutamol - Fluticasone propionate - Lamotrigine - Ondansetron - Pantoprazole sodium sesquihydrate - Polysporin sterile ophthalmic - Oseltamivir phosphate - Paracetamol - Melatonin (5 mg) | - Immunoglobulin human normal, IV - Carbamazepine - Trimethoprim/sulfamethoxazole - Risperidone - Macrogol 3350 - Ergocalciferol - Cetirizine hydrochloride - Salbutamol - Fluticasone propionate - Lamotrigine - Ondansetron - Pantoprazole sodium sesquihydrate - Polysporin sterile ophthalmic - Paracetamol/acetaminophen - Antifungal topical spray - Azelastine hydrochloride/Astelin nasal spray - Sodium chloride - Lidocaine - Ibuprofen - Paracetamol - Oseltamivir phosphate - Lamotrigine - Tolterodine L-tartrate - Levofloxacin - Neomycin/Polymyxin b/ dexamethasone ophthalmic - Epinephrine/lidocaine/tetracaine topical - Montelukast - Ciprofloxacin with hydrocortisone - Docusate sodium - Ergocalciferol - Influenza vaccine - General anesthesia - Supplemental oxygen - Ketamine - Propofol - Cetirizine hydrochloride - Bacitracin with polymyxin B ophthalmic - Cyproheptadine hydrochloride - Alteplase - Mesalazine - Carbamazepine - Immunoglobulin G human, subcutaneous | - Eye infection - Seizure disorder - Asthma - Deafness - Blindness - Developmental delay - Constipation - Gastroesophageal reflux - Dysphagia causing pulmonary aspiration with swallowing - Celiac disease - Snoring - Gastrointestinal bleeding - Irregular sleep-wake cycle - Environmental allergies - Rhinitis - Allergies to diphenhydramine and Varicella Virus Vaccine Live - Lactose intolerance - Urinary incontinence - Meningitis - Intermittent otitis media - Intermittent nausea |
| 3 | <1 | - Pegademase | - Trimethoprim/sulfamethoxazole - Salbutamol sulfate - Amoxicillin/clavulanate - Oxycocet - Guaifenesin | - Trimethoprim/sulfamethoxazole - Salbutamol sulfate - Guaifenesin - Azithromycin - Moxifloxacin hydrochloride - Mometasone furoate - Valaciclovir hydrochloride - Cefalexin - Clindamycin - Vitamin D3 cholecalciferol - Mupirocin - Lidocaine - Lidocaine without epinephrine - Oxycocet - Amoxicillin | - Asthma - Pyloric stenosis - Chronic diarrhea - Herpes zoster |
| 4 | <0.1 | - Pegademase - Exchange transfusion | - Immunoglobulin human normal | - Immunoglobulin human normal, subcutaneous - Azithromycin - Benzocaine menthol lozenge - Vancomycin - Paracetamol/acetaminophen - Enoxaparin - Oxycodone - Alprazolam - Oxcarbazepine - Amoxicillin clavulanate - Alprazolam - Oxcarbazepine - Lidocaine - Midazolam - Paracetamol - Polytrim - Lacosamide - Sofosbuvir/velpatasvir - Amoxicillin and clavulanate | - Cardiovascular accident - Left hemiparesis - Seizure disorder - Chronic hepatitis C - Pityriasis rosea - Pneumococcal meningitis - Pneumococcal bacteremia - Perianal/inguinal anal condyloma - Squamous cell carcinoma - Dysplasia of anus - Bowen’s disease - Common variable immunodeficiency - Urticaria - Recurring chronical renal calculi |
| 5 | 5−6 | - Pegademase - Gene therapy | - Trimethoprim/sulfamethoxazole - Salbutamol sulfate - Budesonide with formoterol fumarate - Immunoglobulin human normal - Supplemental oxygen - Ascorbic acid with biotin and calcium carbonate - Cholecalciferol - Multivitamins - Ibuprofen - Azithromycin - Amlodipine besylate - Omeprazole | - Trimethoprim - Salbutamol sulfate - Budesonide with formoterol fumarate - Supplemental oxygen - Ascorbic acid with biotin and calcium carbonate - Multivitamins - Amlodipine besylate - Omeprazole - Immunoglobulin human normal, subcutaneous - Azithromycin - Ibuprofen - Levofloxacin - Various alimentary tract and metabolism products - Fluconazole - Ceftriaxone - Salbutamol - Prednisone - Amoxicillin and clavulanate Fluticasone propionate - Beclomethasone dipropionate - Ferrous sulfate - Levofloxacin - Paracetamol/acetaminophen - Ceftriaxone - Vancomycin - Piperacillin-tazobactam - Enoxaparin - Oseltamivir - Sodium chloride - Guaifenesin - Sennoside A+B - Ipratropium with salbutamol - Budesonide - Pantoprazole - Probiotic - Acetazolamide - Benzocaine-menthol - Docusate with senna - Immunoglobulins NOS, IV - Aquaphor ointment - Piperacillin tazobactam - Salbutamol - Zolpidem tartrate - Dextrose with sodium chloride and potassium chloride - Fluticasone vilanterol - Simethicone - Ondansetron - Doxycycline | - Allergies to moxifloxacin hydrochloride and ultra-sound gel - Pneumothorax - Asthma - Hypertension - Mild chronic oxyntic gastritis - Chronic lung disease - Near sightedness - Aspergillus - Bilateral tympanostomy tube removal - Anxiety - Anemia - Mastoid bone resection due to necrosis - Vats pleurodesis wedge resection - Pseudomonas pneumonia - Pneumocystis pneumonia |
| 6 | 3 | - Pegademase | - Atovaquone - Lidocaine/prilocaine cream - Epinephrine - Levothyroxine - Salbutamol sulfate - Immunoglobulin G human - Acyclovir - Ibuprofen - Lidocaine and Epinephrine Injection - Triamcinolone acetonide | - Atovaquone - Lidocaine/prilocaine cream - Epinephrine - Levothyroxine - Salbutamol sulfate - Immunoglobulin G human, IM - Acyclovir - Triamcinolone acetonide - Mupirocin - Cefalexin - Clindamycin - acetylsalicylic acid/chlorpheniramine maleate/ phenylephrine bitartrate - macrogol 3350 - Paracetamol/acetaminophen - Miconazole nitrate - Sodium chloride nasal spray - Sodium chloride IV - Sodium chloride nebulizer | - Primary hypothyroidism - Thrombocytopenia - Asthma - Diffuse obstructive pulmonary syndrome - Adrenal insufficiency - Macrocytic anemia - Dermatofibrosarcoma protuberans - Psoriasis - Nausea and vomiting |
| 7 | 0.2 | - Pegademase | - Immunoglobulin NOS, IVIG - Diphenhydramine - Trimethoprim/sulfamethoxazole trimethoprim - Paracetamol/acetaminophen - Azithromycin - Lidocaine - Lactobacillus acidophilus - Epinephrine - Fluorouracil cream - Ibuprofen - Topical tazarotene - Clonidine - Probenecid - Ondansetron - Lamotrigine | - Immunoglobulin NOS, IVIG - Diphenhydramine - Trimethoprim/Sulfamethoxazole trimethoprim - Paracetamol/acetaminophen - Azithromycin - Lidocaine - Lactobacillus acidophilus - Epinephrine - Fluorouracil cream - Lamotrigine - Ibuprofen - Tazarotene - Clonidine - Probenecid - Ondansetron - Dexamethasone - Dexmedetomidine - Diphenhydramine - Fentanyl - Ketamine - Midazolam - Lactic acid cream - Oseltamivir - Cefalexin - Triamcinolone urea, topical - HPV vaccine | - Hepatomegaly and diffuse hepatic stenosis - Severe bilateral plantar warts due to HPV - Moderate hearing loss - Developmental delay - Growth hormone deficiency - 4^th^ and great toe squamous cell carcinoma - ADHD - Hypertension - Anemia - Transaminitis - Hearing loss - Speech and language delays - Oppositional defiant disorder - HPV - Moderate cognitive impairment |

^a^Additional details are available in the Supplemental Text: Individual Patient Narratives

*ADA-SCID,* adenosine deaminase–deficient severe combined immunodeficiency; *ADHD*, attention-deficit hyperactivity disorder; *HPV*, human papilloma virus; *VATS*, video-assisted thoracic surgery

## **Individual Patient Narratives**

### Patient 1:

The male patient was diagnosed 5 months after birth and immediately began receiving pegademase and was on a dose of 43.9 U/Kg split between 2 doses per week at enrollment. The patient was <10 years old with a clinical score of 100 at study enrollment.

The patient experienced moderate injection site pain on day 1 of elapegademase treatment) that resolved in 11 minutes. On day 8 of elapegademase treatment, the patient experienced severe injection-site pain that resolved in 15 minutes. No action was taken with study medication after the first occurrence; the guardian withdrew consent, and the patient was withdrawn from the study after the second occurrence. A subsequent nonclinical investigation by the sponsor found that EDTA in the elapegademase formulation was the likely cause of injection-site pain, and the formulation was revised to remove EDTA. Both occurrences of injection=site pain were evaluated by the sponsor as serious based on meeting the criterion for a medically important serious adverse event (SAE). The patient had no history of injection-site pain and did not experience injection-site pain during the pegademase Lead-In Phase. The pegademase formulation did not contain EDTA.

The patient had low hemoglobin at elapegademase Treatment Phase week 1 and high eosinophil levels (300−400 cells/μL) at Screening, pegademase Lead-In Phase week –1, and elapegademase Treatment Phase week 1 and week 3 (early discontinuation). The patient received concomitant intravenous (IV) normal human immunoglobulin (10 g every 4 weeks) throughout elapegademase therapy.

The patient had both pegademase immunoglobulin G (IgG) and immunoglobulin M (IgM), elapegademase IgM, and anti–polyethylene glycol (PEG) antibodies but no elapegademase IgG antibodies (Fig. 7). Pegademase IgG was detected at the Lead-In Phase at week –1 visit, and at the elapegademase Treatment Phase at week 1 and early discontinuation visit. Pegademase IgM was detected at Screening and all visits, including early discontinuation. Elapegademase IgM was detected at the pegademase Lead-In Phase week –1 visit. Anti-PEG antibodies were detected at the elapegademase Treatment Phase at week 1 and early discontinuation visit. Despite the presence of anti-drug antibodies, trough ADA activity levels for these 2 visits were above 15 mmol/h/L.

### Patient 2:

The male patient was diagnosed 4 months after birth and began pegademase therapy 4 months after diagnosis and was on a dose of 28 U/Kg split between 2 doses per week at enrollment. The patient was 19–30 years old with a clinical score of 40 at study enrollment.

The patient presented to the emergency department after 19.4 months of elapegademase therapy with moderate migraine and associated dehydration and weakness; they had been experiencing worsening headaches and nausea for several months. Intravenous 0.9% sodium chloride infusion was administered, and the patient was admitted overnight and discharged the following day, at which time they were eating and drinking well. Upon discharge, the patient was diagnosed with vestibular migraines. The dehydration, weakness, and headaches were considered resolved.

The patient also experienced skin and genital fungal infection 20 days, influenza 2 months, and external otitis 9.9 months after transitioning to elapegademase; none of these infections were serious or related to the study medication, and all were resolved with appropriate intervention by study end. The patient received concomitant intramuscular (IM) normal human immunoglobulin (20−30 g every morning) throughout elapegademase therapy.

This patient had low hematocrit at pegademase at Lead-In Phase week –1 and elapegademase therapy at weeks 1, 9, 17−112, and 164−177 and low hemoglobin at elapegademase therapy at weeks 1, 17−112, and 164−177.

The patient had mostly positive results for anti-pegademase IgM and anti-elapegademase IgM antibodies from elapegademase Treatment Phase week 10 through Maintenance Phase week 47 (Fig. 7). Pegademase and elapegademase IgG antibodies were detected at elapegademase Treatment Phase week 47. Pegademase IgM antibodies were detected at pegademase Lead-In Phase week –1; elapegademase Treatment weeks 1, 3, and 21; and elapegademase Maintenance Phase week 34. Elapegademase IgM antibodies were detected at Screening and during the elapegademase therapy at weeks 3−34. Anti-PEG antibodies were detected at elapegademase Treatment Phase week 21. Antibody results did not correspond to a change in deoxyadenosine nucleotide (dAXP) or measured adenosine deaminase (ADA) activity. Anti-drug antibodies were not detected after week 47.

### Patient 3:

The male patient was diagnosed ≤10 months after birth and began pegademase therapy at 10 months of age and was on a dose of 30 U/Kg/week at enrollment. The patient was 19–30 years old with a clinical score of 100 at study enrollment.

The patient experienced a groin abscess during the pegademase Lead-In Phase 3 months prior to transition to elapegademase. Additionally, the patient had a groin abscess 28 and 35.8 months, herpes zoster 26.7 months, subcutaneous abscess 31.7 months, and alveolar osteitis 42.4 months after transitioning to elapegademase; all events were nonserious, unrelated to study drug, and resolved. The patient did not receive concomitant immunoglobulin therapy during elapegademase therapy.

The patient had low monocytes at elapegademase Treatment Phase week 1; high neutrophils at Screening, pegademase Lead-In Phase week ‑1, and during elapegademase therapy weeks 1−47 and 99−138; high eosinophils at Screening; low hematocrit at Screening, pegademase Lead-In Phase week -1, elapegademase Treatment Phase week 5−21, elapegademase Maintenance Phase week 34−73, and week 125; low hemoglobin at Screening, pegademase Lead-In Phase week -1, elapegademase Treatment Phase week 5 and 21, and elapegademase Maintenance Phase week 34−73, and week 177−190 and EOS.

The patient had both pegademase and elapegademase IgM, and anti-PEG antibodies (Fig. 7). Pegademase IgM and elapegademase IgM were detected at the pegademase Lead-In Phase week -1 and elapegademase Treatment Phase week 1 and 3. Anti-elapegademase antibodies were detected at the elapegademase Maintenance Phase at week 34.

### Patient 4:

The male patient was diagnosed within a month of birth and underwent exchange transfusions for therapeutic intent, achieving partial immune reconstitution. They discontinued exchange transfusion 12 years after diagnosis. The patient began pegademase therapy at 2 years of age and their dose at enrollment was 30 U/kg split between 2 doses per week. The male patient was 30–40 years old with a clinical score of 100 at study enrollment.

The patient presented to the emergency department after 21.8 months of elapegademase treatment with sore throat, significant pain upon swallowing, fever and chills for the previous 2 days, headaches, palpitations, fluctuance and "pimple" on the roof of the mouth, and right-sided hard palate pain. Physical examination revealed abnormal dentition, dental caries, submandibular edema, and posterior oropharyngeal edema. A computed tomography (CT) scan of the neck/soft tissue showed a large periapical/radicular cyst in the right maxilla associated with the right maxillary cuspid tooth, erosion of the maxillary cortex, and a hard palate mass that may have reflected an extension of odontogenic infection to the hard palate. The next day the patient underwent incision and drainage of the lesion, and the culture of the drainage grew normal oral flora. The patient was started on piperacillin/tazobactam at that time. They were discharged 4 days after being admitted and were prescribed oral amoxicillin/clavulanate potassium 875 mg twice daily for 2 weeks. Six days after being admitted, the patient’s right maxillary cuspid tooth was extracted as an outpatient procedure. The events were assessed as severe, unrelated to study medication, and resolved.

The patient experienced a serious tooth abscess 21.9 months, bacterial conjunctivitis 25.3 months, and upper respiratory tract infection 47 months after transitioning to elapegademase; except as noted, these events were nonserious, unrelated to study drug, and resolved.

The patient had high monocytes at all visits except elapegademase Treatment Phase week 21 and elapegademase Maintenance Phase week 151; high eosinophils at Screening (378 cells/μL) through elapegademase week 17 (range: 315−448 cells/μL) and from elapegademase Maintenance week 34−138 and 164 (range: 305−570 cells/μL); low neutrophils at Screening, pegademase Lead-In Phase week -1, elapegademase Treatment Phase week 1−5, and elapegademase Maintenance Phase week 60, 73, 99, and 138; high hematocrit at Screening, pegademase Lead-In Phase week -1, and elapegademase Treatment Phase week 1−5, and elapegademase Maintenance Phase week 60, and 112−EOS; high hemoglobin at Screening, pegademase Lead-In Phase week -1, and elapegademase Treatment Phase week 5, and elapegademase Maintenance Phase week 60, 112−138, and 203− EOS. The patient received concomitant subcutaneous (SC) human IgG (12.5 g every 2 weeks) throughout elapegademase therapy. Anti-drug antibodies were not detected throughout the study.

### Patient 5:

The female patient was diagnosed approximately 6 years after birth and began pegademase therapy within a year of diagnosis. The patient’s dose of pegademase at enrollment was 30 U/kg/week. The patient underwent gene therapy at approximately 16 years old but failed to achieve immune reconstitution. The patient was 30–40 years old with a clinical score of 100 at study enrollment.

The patient did not meet the inclusion/exclusion criteria at Screening as they were not stable on pegademase dosing for 6 months prior to enrollment due to lack of insurance coverage; however, they were granted a waiver to enter the study. Because of their lack of health insurance, this patient was unable to adhere to a consistent pegademase regimen. The patient subsequently enrolled in a compassionate use program for pegademase therapy for 1 month prior to elapegademase therapy.

At 11.6 months after transitioning to elapegademase therapy, the patient presented to a local hospital with progressive shortness of breath of 2 days’ duration, a productive cough with yellowish sputum, and recurrent fevers. A chest x-ray performed in the emergency room showed findings suspicious for patchy pneumonia, and the patient was hypoxic at 85% on room air, requiring a nonrebreather mask. The patient was started empirically on levofloxacin and piperacillin-tazobactam, given their immunocompromised status. The patient was also started empirically on oseltamivir for possible influenza and on nebulizer treatments and oxygen for respiratory distress and was put on deep vein thrombosis prophylaxis and gastrointestinal prophylaxis. The patient was diagnosed by the infectious disease team with probable influenza A pneumonitis the following day. A day after being admitted, the patient also developed some hemoptysis, which resolved by the third hospital day. Sputum cultures and blood cultures came back negative, and since the patient reported feeling better, they were deemed in stable condition, and antibiotics were discontinued on the fourth hospital day. The patient was discharged in stable condition, at which time the respiratory tract infection was considered resolved. The investigator considered the respiratory tract infection not related to elapegademase therapy, and no action was taken with the study drug.

The patient was again admitted to the hospital after presenting with a cough and bloody sputum that began 19.2 months after transitioning to elapegademase therapy. Due to the hemoptysis, the patient was placed on respiratory isolation. The patient was started on piperacillin-tazobactam, trimethoprim-sulfamethoxazole, and vancomycin. The patient was also treated with albuterol and fluticasone-vilanterol, and was placed on oxygen therapy, which was titrated to keep the minimal partial oxygen pressure (PaO_2_) at 90%. A chest x-ray was performed 3 days after the patient was admitted to the hospital and showed findings suggestive of infection with an atypical etiologic agent. An acid-fast bacilli (AFB) smear and a culture of the patient’s sputum were negative for AFB x3. *Mycobacterium tuberculosis* polymerase chain reaction (PCR) was also negative. A QuantiFERON-TB GOLD test was negative for *M tuberculosis*. A CT scan of the patient’s chest without contrast performed a day after the x-ray revealed moderate multifocal bronchiectasis, mucous plugging, and air trapping. The patient’s symptoms resolved 6 days after they were admitted to the hospital. The investigator considered the hemoptysis unrelated to elapegademase therapy, and no action was taken with the study drug.

After 11 days of elapegademase therapy, the patient experienced a mild gastrointestinal infection unrelated to elapegademase therapy; the infection was resolved at EOS at 125 weeks.

The patient had high monocytes at Screening, pegademase Lead-In Phase week –1, elapegademase Treatment Phase weeks 1−21, elapegademase Maintenance Phase weeks 34−60 and 86, and EOS at week 125; high eosinophils (range: 121−330 cells/μL) at Screening, pegademase Lead-In Phase week –1, elapegademase Treatment Phase weeks 1−9 and 17−21, elapegademase Maintenance Phase weeks 34−60, and EOS at week 125; high platelets at elapegademase Treatment Phase week 17; high neutrophils at Screening, pegademase Lead-In Phase week –1, elapegademase Treatment Phase weeks 1 and 9−13, and elapegademase Maintenance Phase weeks 99−112; low hematocrit at Screening, pegademase Lead-In Phase week –1, elapegademase Treatment Phase weeks 1−21, and elapegademase Maintenance Phase week 34; low hemoglobin at pegademase Lead-In Phase week –1, elapegademase Treatment Phase weeks 1−21, and elapegademase Maintenance Phase week 34. The patient received concomitant SC normal human immunoglobulin (6 g weekly) throughout elapegademase therapy. Anti-drug antibodies were not detected throughout the study.

### Patient 6:

The female patient was diagnosed approximately 3 years after birth and began pegademase therapy soon after. The patient’s dose of pegademase at enrollment was 42.4 U/kg split between two doses per week. The patient was 10–18 years old with a clinical score of 90 at study enrollment.

The patient had high neutrophils at Screening, pegademase Lead-In Phase week –1, elapegademase Treatment Phase weeks 1−9 and 21, and elapegademase Maintenance Phase weeks 34−86; low platelets at elapegademase Treatment Phase weeks 17−21 and elapegademase Maintenance Phase weeks 47 and 73−99. The patient experienced gastroenteritis 2 days prior to transitioning to elapegademase. Additionally, the patient experienced an upper respiratory tract infection 2.7, 7.3, and 21.9 months after transitioning to elapegademase, as well as an earlobe infection 3.7 months, stoma site infection 5.3 months, and vulvovaginal mycotic infection 25.1 months after transitioning to elapegademase. These infectious events were nonserious, unrelated to study drug, and resolved. The patient received concomitant IM human IgG (4/20 kg/L weekly) throughout elapegademase therapy. Anti-drug antibodies were not detected throughout the study.

### Patient 7:

The male patient was diagnosed approximately 2 months after birth and began pegademase therapy within a year after birth. The patient’s dose of pegademase at enrollment was 13 U/kg over 3 or more times per week. The patient was 10–18 years old with a clinical score of 70 at study enrollment.

The patient had low hematocrit at elapegademase Maintenance Phase week 60. They experienced epidermodysplasia verruciformis 1.4 months prior to transitioning to elapegademase; the event was nonserious, unrelated to study drug, and ongoing as of EOS at week 73. The patient received concomitant immunoglobulin, not otherwise specified (dosage unknown), throughout elapegademase treatment. Anti-drug antibodies were not detected throughout the study.

## **Supplemental Text**


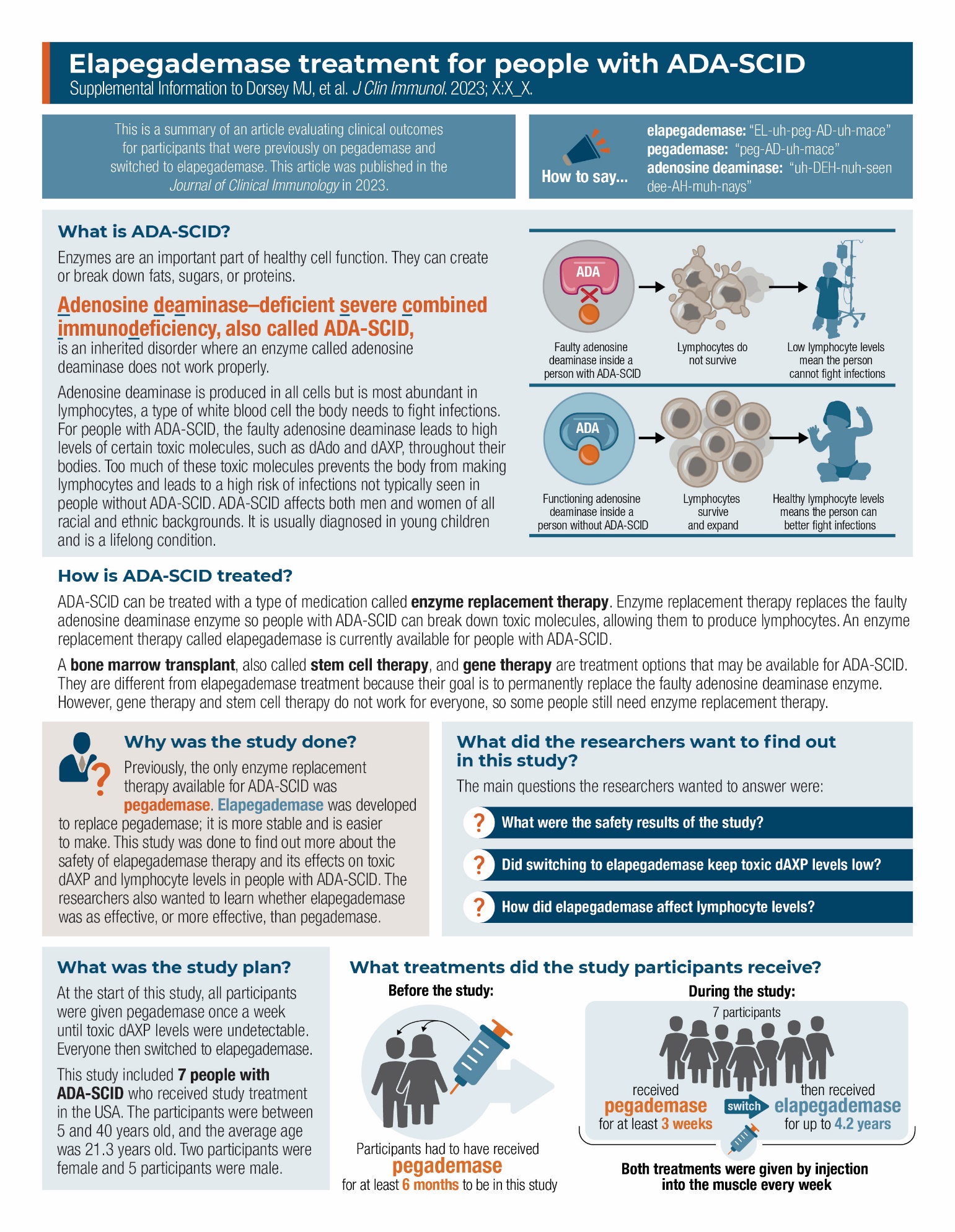


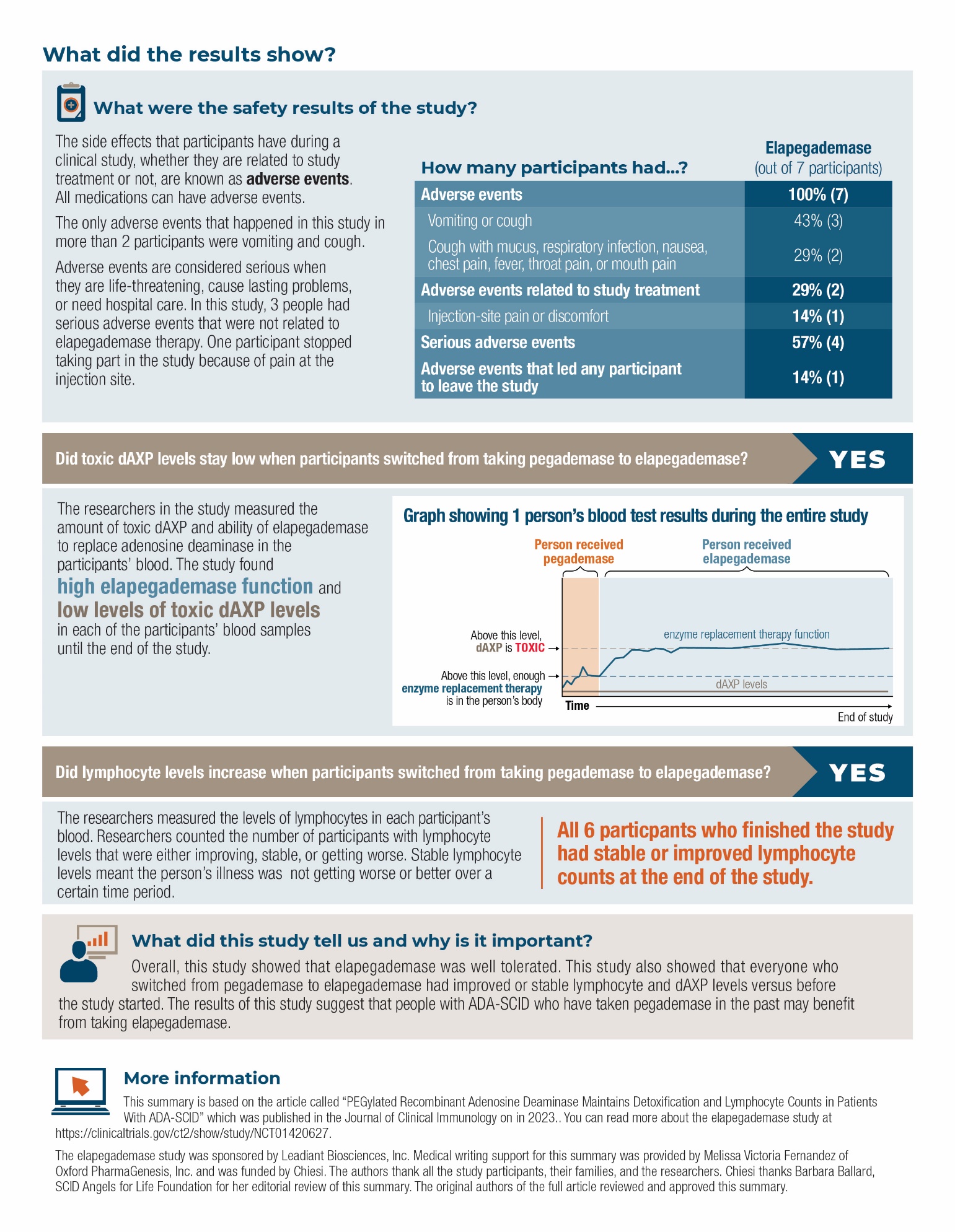

Supplement: Supplementary file 1 — Supplementary file1 (DOCX 1961 KB) [file 10875_2022_1426_MOESM1_ESM.docx]
